# Interferon-Gamma-Inducible Protein-10 (IP-10) and Tumor Necrosis Factor-α (TNF-α) as Serological Predictors of Active Disease Status in Localized Scleroderma

**DOI:** 10.3390/ijms251810134

**Published:** 2024-09-21

**Authors:** Brittany Ashe, Christina Kelsey Zigler, Jonathan Yabes, Kelsey Magee, Katherine Kurzinski, Kathryn S. Torok

**Affiliations:** 1 Department of Pediatrics (Rheumatology), University of Pittsburgh, Pittsburgh, PA 15224, USA; brittany.ashe@chp.edu; 2 Department of Physical Medicine & Rehabilitation, University of Pittsburgh School of Medicine, Pittsburgh, PA 15224, USA; ziglerck3@upmc.edu; 3 Department of Medicine (Biostatistics), University of Pittsburgh, Pittsburgh, PA 15261, USA; jgy2@pitt.edu; 4 Department of Medicine (Psychiatry), University of Pittsburgh, Pittsburgh, PA 15261, USA; mageeke4@upmc.edu; 5 Department of Pediatrics (Nephrology), University of Pittsburgh, Pittsburgh, PA 15224, USA; kurzinskikl5@upmc.edu; 6 University of Pittsburgh Scleroderma Center, Pittsburgh, PA 15261, USA

**Keywords:** localized scleroderma, morphea, biomarker, cytokine, disease activity, interferon-gamma-inducible protein-10, tumor necrosis factor-α

## Abstract

We investigated the ability of a panel of immune-related cytokines and chemokines to predict the disease activity state in localized scleroderma (LS) subjects followed longitudinally. A total of 194 sera samples were obtained from 45 LS subjects with diverse types of LS (40% linear, 20% mixed, 16% craniofacial, 13% generalized, and 11% circumscribed) in our cohort. Cytokines/chemokines that were significantly elevated at the baseline active disease visit compared to the inactive disease state at follow-up were Interferon-Gamma-Inducible Protein (IP)-10 (*p* < 0.021) and Tumor Necrosis Factor (TNF)-α (*p* < 0.033). Mixed effect logit modeling identified IP-10 (Odds Ratio (OR) [95% confidence interval] = 2.1 [1.4, 3.2], *p* < 0.001), TNF-α (OR = 1.8 [1.1, 3.0], *p* = 0.016), and Monocyte Chemoattractant Protein (MCP)-1 (OR = 2.0 [1.1, 3.9], *p* = 0.034) as significant predictors of active disease status. These findings support earlier correlations between IP-10 and TNF-α with disease activity parameters in a cross-sectional Luminex™ serological study and may enhance clinical decision-making when disease activity is challenging to assess by clinical examination alone.

## 1. Introduction

Scleroderma disorders in children encompass both systemic sclerosis (SSc) and localized scleroderma (LS), also termed ‘morphea’. In childhood-onset scleroderma, LS is more common, with an estimated annual incidence of 0.34 per 100,000 children [1]. LS is postulated to have an ‘active’ phase, which is clinically evidenced by erythema and edema and histologically characterized by dense dermal and subcutaneous lymphocytic infiltrate. This is followed by a ‘damage’ phase characterized by dense collagen deposition leading to atrophic patches or linear bands of skin that are thick, hard, and discolored [2]. If inflammation is left unopposed, the fibrosis and resultant atrophy of the skin and underlying connective tissue, including subcutaneous fat, muscle, tendons, and bone, causes deformity and severe functional impairment in actively growing children [3]. Further, in patients with involvement of the scalp and face, in either a linear pattern/grove (en coup de sabre) or hemifacial atrophy pattern (Parry–Romberg syndrome), there is an association with neurological manifestations (20%), including optic neuropathy, strokes, headaches, and seizures [4].

**Figure 1:**
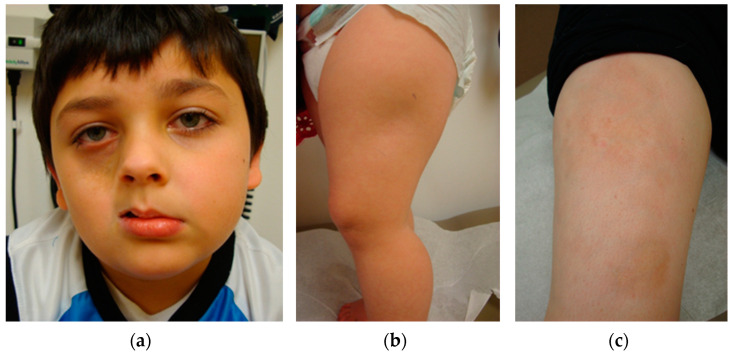
Patients with localized scleroderma subtypes with deep tissue involvement (**a**,**b**) and early active disease (**c**). In these patients, it can be challenging to assess active vs. inactive disease using traditional clinical assessment tools.

The extent and duration of inflammation during the active phase of LS is thought to be the major contributor to long-term disease damage and disability [5,6]; therefore, accurately identifying the active disease state is imperative. However, traditional clinical scoring systems typically rely on superficial features such as erythema and skin thickness which, in certain LS subtypes with deep involvement (Figure 1a,b) and at very early stages of disease flare (Figure 1c), can be difficult to assess. Alternatives or adjuncts to cutaneous assessment include imaging, serologic inflammatory markers, and autoantibody profiling which have their limitations, and ultimately, a skin or deep tissue biopsy is best for activity assessment [5,7,8,9]. However, this approach is not practical in the pediatric setting because it is invasive, painful, and can cause scarring. Consequently, recent work has focused on identifying peripheral blood biomarkers for LS activity.

Previous work in both pediatric and adult LS has identified several cytokines across the T helper (Th)1, Th2, Th17, and macrophage activation-associated pathways, which are elevated in the peripheral blood of LS patients, including Interleukin (IL)-1, IL-2, IL-4, IL-8, IL-13, IL-33, Interferon-Gamma-Inducible Protein (IP-10 (also termed CXCL 10)), and Tumor Necrosis Factor (TNF)-α [10,11,12,13,14,15,16,17]. A more thorough review can be found in Kurzinski and Torok 2011 or Torok et al. 2019 [11,18]. Further studies by our group have demonstrated that several cytokines are elevated serologically in pediatric LS patients compared to healthy controls, namely IP-10, Monocyte Chemoattractant Protein (MCP)1, IL-17a, IL-12p70, Granulocyte Macrophage Colony Stimulating Factor (GM-CSF), Platelet Derived Growth Factor (PDGF)-BB, Interferon (IFN)-a2, and IFN-γ [19]. Within the analysis of these data comparing pediatric-onset LS patients with active disease with those with inactive disease, IP-10 was most strongly correlated with disease activity (FDR *p*-value 0.028). GM-CSF and TNF-α were also noted to correlate specific measures of disease activity. Interestingly, elevations in IP-10 and TNF-α were also noted to correspond to elevations in ESR (FDR *p*-value 0.029). This is noteworthy because IP-10 and TNF-α are macrophage-associated cytokines that promote the differentiation of Th1 lymphocytes [20], and it has been postulated that “active” juvenile-onset LS is primarily a Th1-driven process while quiescent juvenile-onset LS has a pro-fibrotic Th2 signature [21]. More recent work examining the transcriptome present in LS skin has also demonstrated upregulation in several IFN-associated and TNF-α pathways [22,23], opening the possibility that peripheral blood markers may indeed correspond to skin pathology. This study was designed to provide a longitudinal evaluation of cytokine levels as LS disease progresses from active to quiescent to understand if circulating cytokines and chemokines can reflect and predict disease activity state.

## 2. Results

Forty-five pediatric LS patients with three or more sera collections with initial clinical active disease and subsequent inactive disease were analyzed. Demographics can be found in Table 1. The median age at the first sample collection was 11.6 years old. Subsequent blood samples were obtained between 2 and 18 months after the initial visit, with an average of 9.8 months between samples. All patients had at least three sera samples, and 43% had at least five sera samples over an average of 23 months.

Change in activity scores confirm clinical transition from active disease to inactive disease with a median modified Localized Scleroderma Severity Index (mLoSSI) change from 6.0 (IQR 2.5–10.0) to 0.0 (IQR 0–0) and Physician Global Assessment of Disease Activity (PGA-A) from 45 (IQR 25–65) to 0 (IQR 0–0) [24] (Table 2). At the initial sample collection, 13 of 45 (29%) were on systemic therapy; and one (2%) was on topical therapy. All patients were subsequently started on systemic therapy; the majority with methotrexate and corticosteroids (84%), which has been shown to be an effective therapy for patients with localized scleroderma [25,26] and likely accounts for disease remission at follow-up.

From a Luminex panel of 33 cytokines/chemokines, only a few were significantly elevated at the baseline active disease visit compared to an average of all sera collected at the follow-up inactive disease, and include IP-10 (raw *p*-value = 0.021, bootstrap corrected *p*-value = 0.010) and TNF-α (raw *p*-value = 0.033 and bootstrap corrected *p*-value = 0.023). Full results are detailed in Table 3. These findings align with prior data showing that IP-10 is elevated in both blood and tissue samples of patients with localized scleroderma [27]. Additionally, TNF-α has also been implicated as a possible biomarker in active LS [19].

Mixed effect logit modeling was then used to determine which cytokines/chemokines served as predictive markers of disease activity, accounting for the non-independence of observations within a subject over time. Cytokines were log-transformed, and the odds ratio [95% CI] was reported with the Wald *p*-value. IP-10 was shown to be a significant predictor of activity status (OR 2.14 [1.43,3.21], *p* < 0.001) in unadjusted analyses and remained significant when adjusting for all covariates except mLoSSI. This is to be expected, given that mLoSSI is directly related to activity status [28]. Furthermore, MCP-1 (OR 2.02 [1.05, 3.89] *p*-value 0.034) and TNF-α (OR 1.84 [1.12, 3.02] *p*-value 0.016) were also shown to be significant predictors of disease activity in unadjusted analyses. TNF-α was found to be significant when adjusting for all covariates except mLoSSI. MCP-1 was significant for all covariates except mLoSSI and disease duration. Further modeling was performed to analyze the subset of patients on systemic medication at the time of presentation and found that IP-10, TNF-α, and IL-12(p40) were all significant at the *p* < 0.05 level in unadjusted analyses.

## 3. Discussion

This study was designed to evaluate longitudinal cytokine signatures in an attempt to identify functional biomarkers for localized scleroderma (LS). Consistent with previous studies [16,19,27], we demonstrated that IP-10 and TNF-α are inflammatory mediators that are elevated during the active disease state and regress to normal values during quiescent disease. Furthermore, using statistical modeling, we identified that both IP-10 and TNF-α are predictive biomarkers of active disease status. These findings suggest that IP-10 and TNF-α could enhance clinical decision-making, particularly in cases where clinical examination alone is insufficient to determine disease activity.

Limitations to our work include a small sample size, which could lead to statistical error. However, the rare incidence of localized scleroderma makes this difficult to mitigate. Additionally, about one-third of our patients were on systemic immunosuppressive therapy at the time of initial sample collection, which could skew the initial cytokine profile. It would be unethical to withhold treatment until sample collection, so we sought to mitigate this effect by collecting samples only from patients whose disease was deemed to be active by objective measures (mLoSSI and PGA) despite treatment initiation. It should be noted that two-thirds of patients were not on systemic therapy at the time of initial sample collection. Interestingly, IL-6 was not noted to be significantly elevated, nor was there a significant difference between active and inactive disease states. This stands in contrast to prior reports suggesting that tocilizumab (a monoclonal antibody against IL-6) can be effective in refractory LS [29]. However, even in disease states for which IL-6 blockade is the standard of care (polyarticular juvenile idiopathic acrthritis (JIA), systemic onset JIA), IL-6 levels in sera samples obtained from patients with active disease are not always elevated [30]. This suggests that pathogenesis may be related to the complex interplay between cytokines and patterns of cytokines rather than directly due to the effects of a singular cytokine, though this does not eliminate the role of single cytokines as biomarkers.

Interferon-Gamma-Inducible Protein-10 (IP-10), also known as C-X-C motif chemokine ligand (CXCL)10, is an inflammatory chemokine that has been implicated in the pathogenesis of several autoimmune diseases, such as systemic lupus erythematosus (SLE) [31], juvenile dermatomyositis (JDM), and systemic sclerosis (SSc) [32]. Both keratinocytes and immune cells such as neutrophils, eosinophils, monocytes, and macrophages secrete IP-10 after stimulation by IFN-ɣ. IP-10 then acts through C-X-C Motif Chemokine Receptor (CXCR)-3 receptors to attract Th1 lymphocytes, macrophages, and natural killer (NK) cells to local sites of inflammation. These immune cells can then release more IFN-ɣ and TNF-α, perpetuating the immune process [33].

Prior work by our group has implicated IP-10 in the pathogenesis of LS through evidence such as intense IP-10 staining at sites of cellular infiltration in the dermis of LS skin and a strong correlation of disease activity parameters with peripheral blood cytokine levels [27]. Further, single-cell transcriptomics work has confirmed that CXCR3-related cytokines, including *CXCL9* and *CXCL10*, are upregulated in morphea skin [34]. Similarly, other groups, including Mertens et al., have demonstrated increased levels of *CXCL10* gene expression in active morphea tissue biopsies [35]. Their group also showed that elevated serum levels of IP-10 are associated with active disease as defined by mLoSSI [36]. This was also confirmed in an international cohort of Turkish LS and SSc patients [37]. The significant elevation of IP-10 in the sera and tissue of LS patients with active versus inactive disease, along with correlations between IP-10 levels and standardized disease outcome measures of activity in LS, strongly suggest that IP-10 may be a biomarker for disease activity in LS.

Further, CXCR3-related cytokines have been shown to be necessary for the development of scleroderma. In a bleomycin-induced mouse model of scleroderma, with a shorter duration of bleomycin induction meant to simulate localized scleroderma/morphea, CXCL9 and CXCR3 knockout mice were protected from the development of fibrosis. CXCL10 knockout mice were not as sheltered from the development of fibrosis but displayed a partial protective role. The investigators further studied the effect of CXCL9 signaling by culturing healthy CD45−CD31 fibroblasts with recombinant CXCL9 leading to upregulation of *Col1a1*, the gene that encodes type 1 collagen [38] providing a direct link to the development of skin fibrosis.

TNF-α is a pro-inflammatory cytokine secreted by activated macrophages, T-lymphocytes, and natural killer cells, implicated in a spectrum of autoimmune diseases from rheumatoid arthritis to inflammatory bowel disease to uveitis [39]. While it has wide-ranging immunomodulatory effects, its ability to induce fibroblast growth and collagen synthesis is particularly relevant to scleroderma [40]. Hasegawa et al. have shown that serum levels of TNF-α are significantly elevated in patients with LS compared to healthy controls. Furthermore, TNF levels were highest in the patients with the shortest disease duration, suggesting a potential role in the pathogenesis of LS [15] and a possible avenue for therapeutic intervention.

TNF-α has also been implicated in the pathogenesis of scleroderma. Murota et al. performed similar in vivo experiments with wild-type (WT) and TNF-receptor deficient mice using the bleomycin model of scleroderma. They noted that the TNF-receptor-deficient mice developed skin sclerosis earlier than WT mice, and MMP-1, an anti-fibrotic protease, was significantly inhibited [41]. Further, while the exact mechanism by which MTX exerts its anti-inflammatory effect is incompletely understood, its primary mechanism is to inhibit the synthesis of pyrimidines. As a secondary effect, MTX has also been shown to both directly and indirectly inhibit TNF-α [42]. That treatment with MTX is sufficient to control disease activity in many patients also suggests that TNF-α likely plays a role in the pathogenesis of scleroderma.

In 2012, The Childhood Arthritis and Rheumatology Research Alliance (CARRA) released guidelines for the treatment of localized scleroderma, which include a combination of methotrexate and corticosteroids [43]. Patients who fail first-line therapy or who experience intolerable side effects may be offered mycophenolate mofetil (MMF), but this also has the undesirable side effect of reversible bone marrow suppression, which may necessitate treatment interruptions or discontinuation. Ferguson and colleagues described the case of a 14-year-old female with progressive mixed morphea covering 80% of her body surface area, who demonstrated only a partial response to standard therapy and an intolerable side effect profile on MMF [44]. Treatment with infliximab, a TNF-α antagonist, was considered given several case reports within the adult literature that demonstrate a favorable effect on disease progression [45]. Within 9 months of the initiation of infliximab therapy (with leflunomide to prevent the formation of neutralizing antibodies), she showed significant improvement in both patient and physician measures of disease burden. Similarly, it has been reported in the adult systemic sclerosis literature that treatment with infliximab has been shown to improve several markers of disease activity [46]. These case reports, along with evidence of IP-10 and TNF-α as biomarkers of disease activity, represent an important avenue for further translational research.

## 4. Materials and Methods

### 4.1. Study Participants

The University of Pittsburgh Institutional Review Board approved blood sample and clinical data collection of LS patients through the National Registry of Childhood Onset Scleroderma (NRCOS) protocol, which has been approved by the University of Pittsburgh IRB since 2003 (the University of Pittsburgh #PRO11060222). Consent was obtained from parents for their children’s participation in the NRCOS study, and assent was obtained when appropriate. All LS patients included in this study had clinic visits from 2003 to 2013, where standardized demographic, clinical, and laboratory data were collected. Research blood samples were obtained from subjects by venipuncture at routine clinical visits as part of their participation in the pediatric scleroderma registry. Sera were separated from whole blood via density-gradient centrifugation at 4 °C within 4 h of blood collection and promptly stored in 200 μL aliquots at −80 °C storage until experimentation.

At the concurrent visit in which blood was obtained, standardized demographic and clinical LS outcome measures were collected. The extent and activity of LS was determined by the treating physician (KST) using two validated measures of clinical activity, the Physician Global Assessment of Disease Activity (PGA-A) and the modified Localized Scleroderma Severity Index (mLoSSI) [28]. The mLoSSI assesses disease activity through three different physical exam findings summed across multiple body sites: erythema, skin thickness, and the presence of new/expanding lesions. The PGA-A similarly assesses disease activity by assigning the overall activity status of the disease (cutaneous and non-cutaneous) on a 100 mm visual analog scale. Active disease was defined as both the PGA-A and mLoSSI being greater than zero. Validated disease damage outcome parameters that were collected concurrently included the Localized Scleroderma Damage Index (LoSDI), a cutaneous measure capturing skin dyspigmentation, dermal atrophy, and subcutaneous atrophy, and the Physician Global Assessment of Disease Damage (PGA-D), measuring cutaneous and non-cutaneous overall disease damage status.

### 4.2. Cytokine Measurement

Sera cytokines and chemokines were measured using the Luminex™ bead immunoassay system (BioRad, Hercules, CA, USA), according to the manufacturer’s instructions at the University of Pittsburgh Cancer Institute (UPCI). The plasma samples were undiluted and compared to a High PMT-Standard Dilution Series (BioRad, Hercules, CA, USA). Samples were run in duplicate to ensure reliability. Inter-panel and intra-assay control plasma samples were included to ensure consistency across panels. The cytokines and chemokines included in this panel were Th1-associated cytokines: IL-2, IL-12(p40), IL-12(p70), IL-27, IL-28A, IFN-ɣ; Th2-associated cytokines: IL-1Ra, IL-3, IL-4. IL-5, IL-10, IL-13, IL-17E (IL-25), IL-31, IL-33; TH17-associated cytokines: IL-1b, IL-6, IL-17A, IL-17F, IL-22, IL-23; macrophage-associated cyto/chemokines: IFN-2a, IL-8, IP-10, MCP-1, MIP-1, TNF-α, GM-CSF; and vascular markers: VEGF, EGF, and Eotaxin.

### 4.3. Data Analyses

All analyses were performed using SPSS v. 20 (SPSS, Chicago, IL, USA). The mean or median was used to describe data where appropriate.

LS subjects with three or more sera samples with initial blood drawn during the active disease state and subsequent blood samples during the inactive disease state were analyzed. The initial comparison involved baseline active disease samples compared with subsequent inactive disease samples (subsequent inactive sample cytokines were averaged), with *p*-value corrections for multiple comparisons. Log-ranking was used to account for left-censored data, and bootstrapping (BS) was employed to correct for potential within-subject correlations and to obtain robust solutions. Subsequently, mixed effect logit models were used to predict active disease status, with cytokine/chemokine as the predictor, including unadjusted (cytokine alone as the predictor) and adjusted analyses (cytokine plus biological covariate). Covariates included age at first sample, disease duration, medication status, gender, modified Localized Scleroderma Skin Severity Index (mLoSSI), Localized Scleroderma Skin Damage Index (LoSDI), anti-nuclear antibody (ANA), anti-single stranded DNA (anti-ssDNA), and anti-histone (AHA) antibodies. Cytokines were log-transformed, and the OR [95% CI] was reported with Wald *p*-value, with significance being <0.05.

## 5. Conclusions

In summary, this study highlights the significance of IP-10 and TNF-α as potential biomarkers for disease activity in localized scleroderma. Both cytokines were found to be elevated in the active disease state and reduced during quiescent periods in LS patients, consistent with their known roles in autoimmune and inflammatory pathways. The predictive value of IP-10 and TNF-α for active disease, demonstrated through statistical modeling, underscores their potential utility in augmenting clinical decision-making, particularly in ambiguous cases. The involvement of IP-10 in attracting immune cells to sites of inflammation and its upregulation in morphea skin emphasizes its role in the pathogenesis of LS. Similarly, the ability of TNF-α to induce fibroblast growth and collagen synthesis, along with its elevated levels in early disease stages, suggests it is a critical player in LS progression and a potential target for therapeutic intervention. Case reports of successful TNF-α antagonist treatment in patients with refractory disease further support this therapeutic avenue. Collectively, these findings advocate for further translational research to validate and refine the use of IP-10 and TNF-α as biomarkers and therapeutic targets, ultimately aiming to improve patient outcomes in localized scleroderma.

## Figures and Tables

**Table 1:** LS subjects’ (n = 45) baseline demographic and clinical variables.

	LS Subjects
**Gender**, n (%)	
Male	16 (35.6)
Female	29 (64.4)
**Caucasian**, n (%)	39 (86.7)
**Subtype**, n (%)	
Linear-trunk and extremity involvement	18 (40.0)
Mixed	9 (20.0)
Linear Head involvement	7 (15.6)
Generalized Morphea	6 (13.3)
Circumscribed Morphea—Deep	3 (6.7)
Circumscribed Morphea—Superficial	2 (4.4)
**Number of longitudinal visits with sera collected and analyzed**	
Three	11
Four	15
Five	17
Six	0
Seven	0
Eight	2
**Extracutaneous manifestations**, n (%)	
Musculoskeletal	29 (64)
Joint contracture	13 (29)
Arthritis	3 (7)
Myalgia	4 (9)
Neurologic	17 (38)
Ophthalmologic	8 (18)
**Auto-antibodies**, n (%)	
Anti-nuclear antibody	14 (31)
Anti-ssDNA antibody	11 (24)
Anti-histone antibody	14 (31)
**Abnormal labs (elevated)**, n (%)	
Aldolase	13 (29)
CPK	6 (13)
WBC	9 (20)
**Medications at first sample**, n (%)	
None	23 (51.1)
Methotrexate	2 (4.4)
Prednisone	0 (0.0)
Methotrexate + Prednisone	11 (24.4)
Topical	6 (13.3)
Doxycycline	1 (2.2)
Methotrexate + Prednisone + Topical	1 (2.2)
Methotrexate + Topical	1 (2.2)
	**Median (IQR)**
Age at onset (years)	8.0 (5.5–10.4)
Age at 1st sample collection (years)	11.6 (9.1–14.7)
Time from sample 1 to sample 2 (months)	6.8 (3.4–17.5)
Disease duration at first visit (years)	2.9 (0.9–5.4)
Average duration of follow-up (years)	1.6 (0.9–2.0)

**Table 2:** Clinical measures of disease activity and damage by visit.

Clinical Measures	Median (IQR)
First Visit	
mLoSSI	6.0 (2.5–10.0)
PGA-A	45.0 (25.0–66.5)
LoSDI	10.0 (7.0–20.0)
PGA-D	36.0 (30.0–45.0)
Second Visit	
mLoSSI	0.0 (0.0–2.0)
PGA-A	0.0 (0.0–6.0)
LoSDI	7.0 (5.0–13.0)
PGA-D	29.0 (17.5–40.0)
Last Visit	
mLoSSI	0.0 (0.0–0.0)
PGA-A	0.0 (0.0–0.0)
LoSDI	7.0 (4.0–14.5)
PGA-D	28.0 (12.0–33.0)

Abbreviations: mLoSSI: modified Localized Scleroderma Skin Index; LoSDI: Localized Scleroderma Damage Index; PGA: Physician Global Assessment; -A: activity; -D: damage.

**Table 3:** Average concentration of cytokine in sera samples by disease activity status.

Cytokine	Active Median (IQR)	Inactive Median (IQR)	*p*-Value	BS-Corrected *p*-Value
EGF:	163.65 (99.25, 344.28)	207.34 (134.92, 263.50)	0.393	0.383
Eotaxin:	63.14 (43.92, 75.82)	67.19 (51.63, 83.61)	0.446	0.467
GM-CSF:	0.47 (0, 2.38)	0.74 (0.12, 1.60)	0.735	0.773
IFNa2:	3.67 (0, 32.64)	5.31 (0, 18.26)	0.938	0.940
IL-10:	1.28 (0, 4.39)	0.99 (0.13, 2.78)	0.854	0.850
IL-12(p40):	0 (0, 12.06)	0 (0, 4.99)	0.591	0.640
IL-12(p70):	0 (0, 1.32)	0.13 (0, 0.78)	0.707	0.680
IL-13:	0 (0, 11.88)	0.20 (0, 6.62)	0.501	0.530
IL-17A:	0 (0, 0.74)	0 (0, 0.34)	0.912	0.907
IL-17E/IL-25:	22.50 (16.00, 32.00)	23.38 (20.10, 29.00)	0.862	0.833
IL-17F:	57.80 (29.20, 81.00)	57.70 (37.70, 87.65)	0.837	0.840
IL-1a:	0 (0, 17.74)	0.69 (0, 16.29)	0.521	0.530
IL-1b:	0 (0, 0.78)	0 (0, 0.67)	0.954	0.943
IL-1Ra:	15.31 (3.82, 29.05)	13.49 (6.50, 20.55)	0.552	0.577
IL-2:	0 (0, 1.11)	0.01 (0, 0.33)	0.916	0.907
IL-21:	23.50 (19.50, 32.00)	25.38 (20.00, 32.77)	0.844	0.873
IL-22:	40.00 (26.50, 68.80)	40.70 (31.00, 66.75)	0.907	0.930
IL-23:	22.50 (15.80, 42.00)	23.50 (18.05, 44.00)	0.753	0.757
IL-27:	74.50 (55.50, 130.50)	78.03 (58.28, 138.27)	0.615	0.617
IL-28A:	22.50 (18.00, 33.00)	22.75 (18.83, 32.20)	0.926	0.930
IL-3:	0 (0, 0)	0 (0, 0)	0.633	0.477
IL-31:	28.50 (19.00, 67.50)	34.80 (21.38, 71.57)	0.614	0.600
IL-33:	29.50 (21.50, 53.50)	32.27 (23.88, 54.50)	0.702	0.703
IL-4:	0 (0, 21.66)	0 (0, 13.62)	0.926	0.940
IL-5:	0.13 (0, 0.76)	0.10 (0, 0.42)	0.578	0.600
IL-6:	0 (0, 1.25)	0 (0,0.79)	0.891	0.853
IL-8:	7.32 (3.09, 11.79)	4.90 (3.58, 11.03)	0.984	0.973
INF-y:	1.06 (0.27, 5.32)	1.36 (0.46, 3.77)	0.834	0.880
**IP-10:**	**338.48 (249.39, 606.23)**	**243.55 (207.35, 369.65)**	0.021 *	0.010 *
MCP-1:	331.00 (245.31, 425.64)	288.18 (220.18, 397.51)	0.107	0.083
MIP-1a:	4.94 (1.89, 9.17)	4.22 (1.94, 6.88)	0.548	0.543
**TNF-a:**	**5.54 (3.77, 8.49)**	**4.49 (3.22, 5.69)**	0.033 *	0.023 *
VEGF:	136.38 (72.26, 280.38)	124.02 (38.25, 227.93)	0.292	0.313

* signifies significant *p*-value, which is bolded in the Table.

## Data Availability

Details of raw data will be available upon request. Please contact the corresponding author.
